# Supplementary material for: In Situ Visual Distribution of Gelsemine, Koumine, and Gelsenicine by MSI in Gelsemium elegans at Different Growth Stages
Source: Molecules. 2022 Mar 10;27(6):1810. doi: 10.3390/molecules27061810 (PMC8952314; doi:10.3390/molecules27061810)
Supplement: Supplementary file 1 [file molecules-27-01810-s001.zip › molecules-1602686-supplementary.pdf]

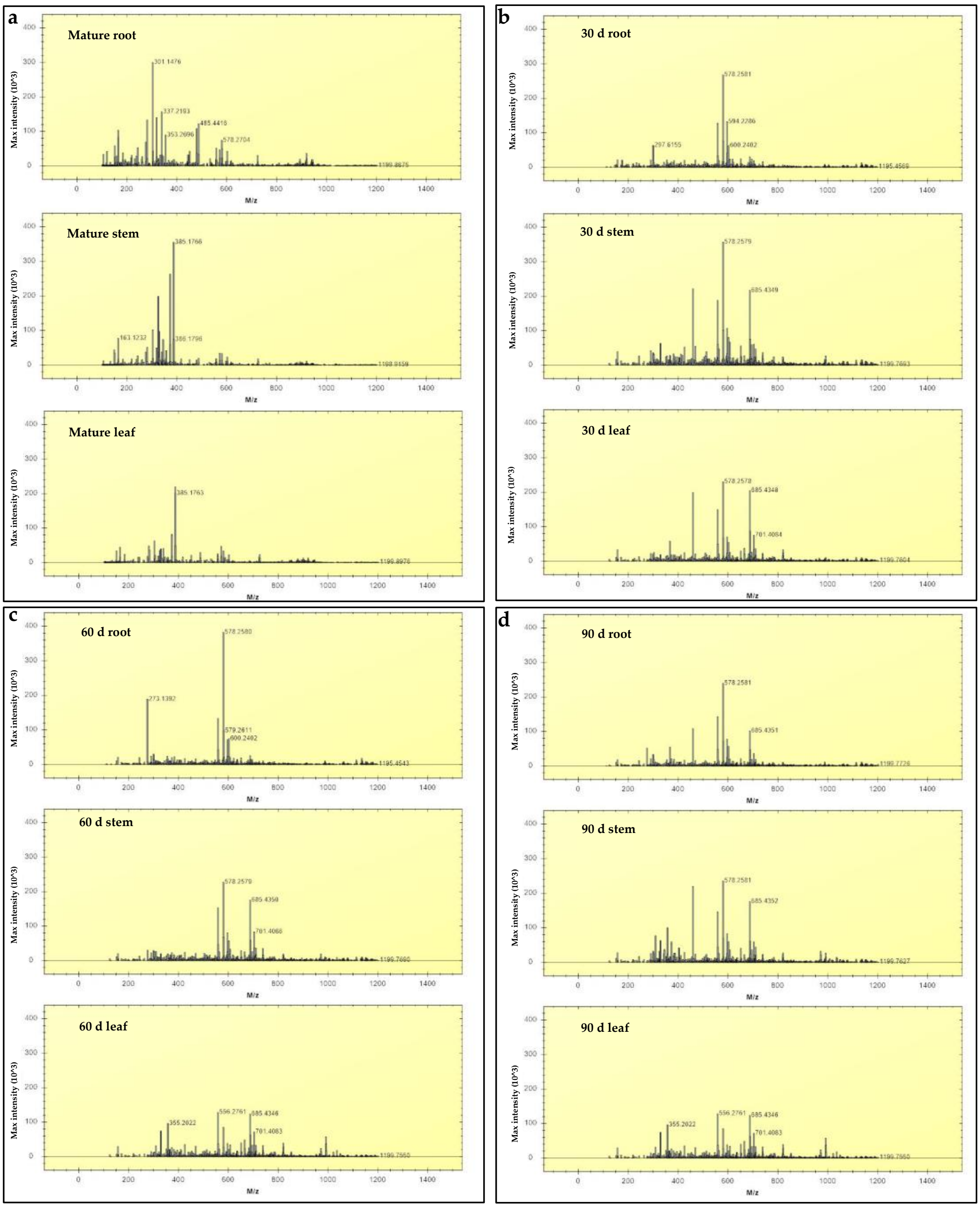

Figure S1. Ion mass spectrums of DESI-MSI. The mature stage (**a**) and 30 d (**b**), 60 d (**c**), 90 d (**d**) old seedling of *G. elegans*.

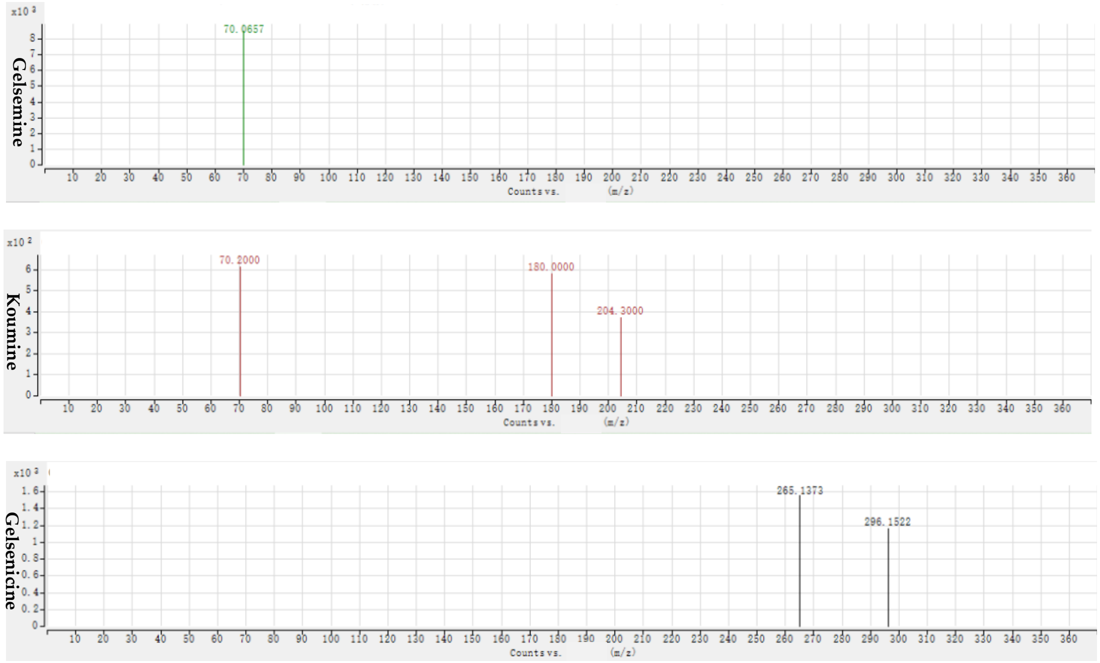

Figure S2. Ion mass spectrums of gelsemine, koumine and gelsenicine by LC-MS/MS.

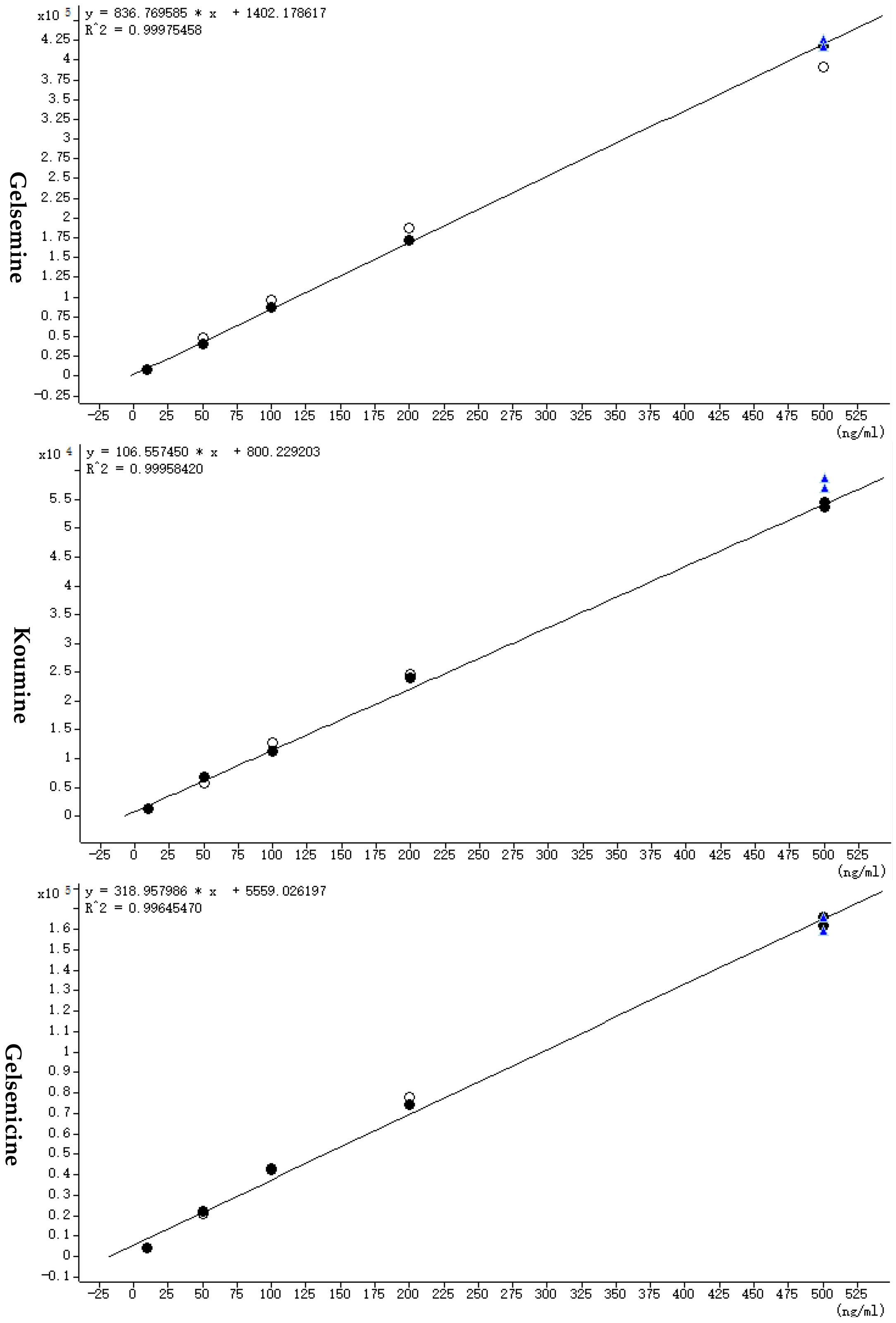

Figure S3. The standard curves of gelsemine, koumine and gelsenicine by LC-MS/MS.
